# In-Depth Characterization of Monocyte-Derived Macrophages using a Mass Cytometry-Based Phagocytosis Assay

**DOI:** 10.1038/s41598-018-38127-9

**Published:** 2019-02-13

**Authors:** Daniel Schulz, Yannik Severin, Vito Riccardo Tomaso Zanotelli, Bernd Bodenmiller

**Affiliations:** 1 Institute of Molecular Life Sciences, University of Zürich, Winterthurerstrasse 190, 8057 Zürich, Switzerland; 2 Department of Biology, Institute of Molecular System Biology, Swiss Federal Institute of Technology (ETH Zürich), Otto-Stern-Weg 3, Zürich, Switzerland; 3 Systems Biology PhD Program, Life Science Zürich Graduate School, ETH Zürich and University of Zürich, 8057 Zürich, Switzerland

## Abstract

Phagocytosis is a process in which target cells or particles are engulfed and taken up by other cells, typically professional phagocytes; this process is crucial in many physiological processes and disease states. The detection of targets for phagocytosis is directed by a complex repertoire of cell surface receptors. Pattern recognition receptors directly detect targets for binding and uptake, while opsonic and complement receptors detect objects coated by soluble factors. However, the importance of single and combinatorial surface marker expression across different phenotypes of professional phagocytes is not known. Here we developed a novel mass cytometry-based phagocytosis assay that enables the simultaneous detection of phagocytic events in combination with up to 40 other protein markers. We applied this assay to distinct monocyte derived macrophage (MDM) populations and found that prototypic M2-like MDMs phagocytose more *E. coli* than M1-like MDMs. Surface markers such as CD14, CD206, and CD163 rendered macrophages phagocytosis competent, but only CD209 directly correlated with the amount of particle uptake. Similarly, M2-like MDMs also phagocytosed more cancer cells than M1-like MDMs but, unlike M1-like MDMs, were insensitive to anti-CD47 opsonization. Our approach facilitates the simultaneous study of single-cell phenotypes, phagocytic activity, signaling and transcriptional events in complex cell mixtures.

## Introduction

Professional phagocytes, including neutrophils, macrophages, and dendritic cells, mediate the internalization and killing of microorganisms, a process crucial to the innate immune response. Phagocytosis is also important in the adaptive immune response^1^, tissue remodeling^2^, wound healing^3–5^, and tissue homeostasis^6,7^. Resistance to phagocytosis is associated with tumor promotion and progression and other disease states^8,9^. Hence, a better understanding of phagocytosis and phagocytic cells could facilitate the development of novel therapeutic approaches.

Phagocytes recognize and differentiate between highly heterogeneous target particles via a vast repertoire of receptors^10^. Pattern recognition receptors bind directly to epitopes on target particles such as the conserved motifs of bacterial pathogens^11^, whereas opsonic receptors and complement receptors trigger internalization indirectly via the recognition of opsonins, which are soluble molecules (e.g., antibodies) that selectively bind to foreign particles^12^.

Not all phagocytes possess the same arsenal of receptors, and the same type of phagocyte may express different receptors depending on the physiological niche. Macrophages in particular stand out due to their “phenotypic plasticity”, their ability to adapt receptor expression to the tissue microenvironment^13^. Traditionally, the system for macrophage classification has been a continuous spectrum from the pro-inflammatory M1-like to the anti-inflammatory M2-like^14^ which has recently been shown to be a strong simplification of the *in vivo* situation in which tissue macrophages display a vast phenotype complexity^15–18^.

Developments in mass cytometry, a technique that combines flow cytometry with mass spectrometry, have enabled detection of up to 40 protein readouts in single cells^19,20^. This has facilitated the understanding of phenotypic diversity of macrophages found in mouse and human *in vitro* and *in vivo*^16,21–24^. These studies have identified the phenotypic signatures of macrophages found in different types of tissues and at different stages of cancer development with an unprecedented level of resolution. However, due to the absence of a functional readout that can be assessed using this approach, previous analyses were unable to directly link high-dimensional phenotype to function.

Different macrophage phenotypes have different phagocytic activities^25–29^. Hence, linking phagocytic behavior to an in-depth marker-based characterization of macrophage subpopulations will result in a more informative characterization of macrophage plasticity. Here, we present a functional assay to assess phagocytic activity by mass cytometry. This newly developed method combines an in-depth phenotypic characterization of macrophages based on expression of 36 protein markers with an analysis of biological function. We demonstrate the relevance of this method by assessing the abilities of macrophages activated *in vitro* under 10 different conditions to phagocytose bacteria and cancer cells. By correlating the phagocytosis activity with marker expression of individual cells, we defined marker signatures preferentially associated with phagocytosis of particular targets. Our mass cytometry-based assay can be used to link cell phenotype to phagocytotic function in phagocytes in health and disease and further allows the evaluation of signaling responses in phagocytes upon ingestion of different targets.

## Results

### Development of a novel mass-cytometry-based phagocytosis assay

To make phagocytic events detectable by mass cytometry, we established a protocol for metal-based staining of target cells based on either osmium or ruthenium tetroxide. Both reagents are highly reactive with lipids and aromatic compounds. Neither osmium nor ruthenium are present in biological samples, and their masses lie within the detection range of mass cytometry instruments^30^. Moreover, these metals are detected on the two opposite ends of the mass range (98–104 for Ru and 184–192 for Os), and therefore assay optimization for both isotopes allow for more user-defined options.

**Figure 1:**
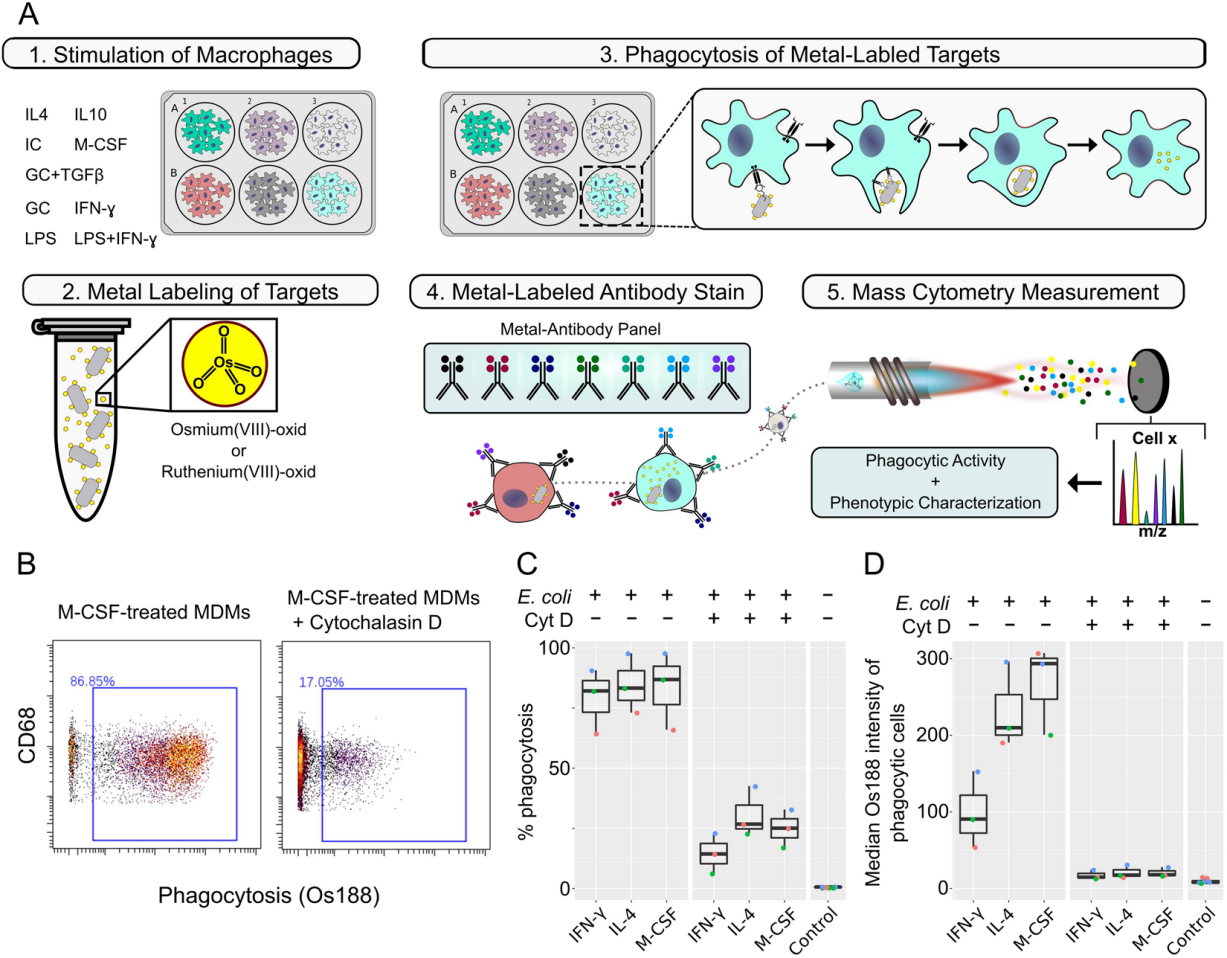
Mass cytometry-based phagocytosis assay of *E. coli* target cells. (**A**) Schematic of the mass cytometry-based phagocytosis assay. (**B**) Scatterplots from M-CSF-stimulated MDMs incubated with OsO_4_-labeled *E. coli* for 60 min with or without cytochalasin D, which was added 10 min prior to *E. coli* cell addition. Phagocytosis was determined based on a global, manually defined gate for ^188^Os intensity. (**C**) Boxplot of the percentage of MDMs stimulated as indicated that had phagocytosed labeled *E. coli* cells after 60 min. No *E. coli* cells were added to the control samples. (**D**) Boxplot of median ^188^Os intensity in MDMs stimulated as indicated in the phagocytic-positive gate. Assays were conducted with three biological replicates (indicated by color).

To initiate phagocytosis, monocyte-derived macrophages (MDMs), generated upon M-CSF treatment of monocytes, were incubated with metal-labeled target cells. After incubation, the MDMs were harvested and stained with antibodies (Material and Methods). Data were acquired on a mass cytometer (Fig. 1A). A gating strategy was used to identify MDMs that had undergone phagocytosis and to exclude debris, dead cells, and non-differentiated monocytes (Fig. S1).

### Phagocytic affinity and capacity

To validate that our assay detects phagocytic events, we made use of cytochalasin D, an inhibitor of actin polymerization that has been used for decades to block phagocytosis through inhibition of actin polymerization^31,32^. We monitored phagocytosis of osmium-stained *Escherichia coli* (*E. coli*) DH5-*α* cells by M-CSF-treated MDMs with and without prior cytochalasin D treatment. MDMs and target cells were incubated together for 60 minutes. We observed a strong decrease in phagocytosis upon cytochalasin D treatment (Fig. 1B), confirming that our assay detected phagocytic events. Of note, this assay does not distinguish between initial steps in phagocytosis, like the binding of particles and particles already internalized. We then determined the percentages of IFN-γ-treated cells (prototypic M1-like MDMs) and IL-4-treated cells (prototypic M2-like MDMs) that had phagocytosed target cells (Fig. 1C). More than 60% of MDMs were phagocytic positive independent of their polarization condition. Interestingly, of the phagocytic-positive MDMs, IFN-γ-treated MDMs had engulfed 2-fold fewer particles than IL-4-treated or M-CSF-only treated MDMs (Fig. 1D). Similar results were obtained for *E. coli* cells stained with ruthenium tetroxide (Fig. S2) Thus, we were able to distinguish between phagocytic affinity (percentage of phagocytic cells) and phagocytic capacity (amount of bound/internalized target cells in phagocytic positive cells).

To investigate differences in phagocytic affinity and capacity among differently stimulated MDMs, we investigated different MDM to target cell ratios (MDM/T). We observed that under low ratios (1/10) different phagocytic affinities became apparent that could not be detected under high target cell ratios, whereas differences in phagocytic capacity became more apparent under high ratios (Fig. S3). The ratio experiments were performed after 30-minute incubation with *E. coli*. Because these results did not differ from experiments in which MDMs and target cells were incubated for 1 hour (Fig. S3, compare 1/50 ratio to Fig. 1C and D), subsequent experiments were performed with 30-minute incubation times. From these experiments, we concluded that the assay presented assesses phagocytic affinity and capacity by mass cytometry and simultaneously allows for the characterization of macrophage phenotypes.

### M2-like MDMs show higher phagocytic affinities and capacities than M1-like MDMs

**Figure 2:**
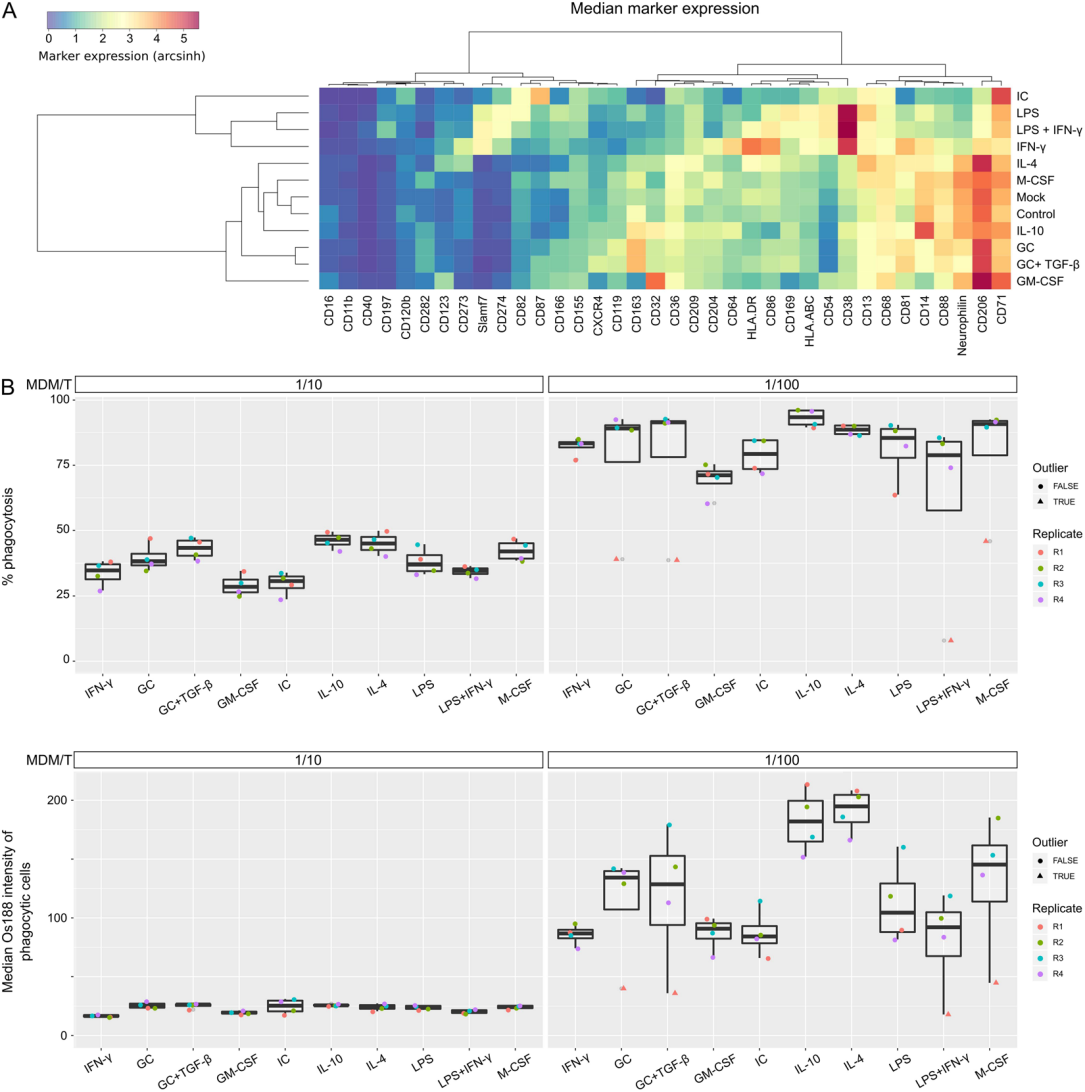
Marker expression and phagocytic activity of stimulated MDM populations. (**A**) The median arcsinh transformed marker intensity is shown for the ten differently stimulated MDM populations of the 1:100 (MDM/T) condition. Control MDMs were not treated with *E. coli* and were not further polarized after 5 days. Mock represents MDMs that were not further polarized after 5 days but were treated with *E. coli*. (**B**) The median percent phagocytic cells (top) and the median ^188^Os intensity (bottom) are shown for MDM to target cell ratios of 1/10 (left) and 1/100 (right). Dots are colored according to replicate. Data points defined as outliers are highlighted as triangles. All assays were conducted with three biological replicates (R1, R2, R4) and a technical replicate of R2 (R3).

The initial method development experiments revealed different phagocytic affinities and capacities between three differently polarized MDM populations, suggesting functional differences in polarized macrophage populations. Therefore, we next performed a comprehensive phagocytic characterization of M-CSF-treated MDMs polarized for 24 hours with eight different stimuli (IL-4, IL-10, IC, GC, GC + TGF-β, LPS, LPS + IFN-γ, and IFN-γ) chosen according to experimental standards proposed by Murray *et al*.^33^ as well as control cells treated with GM-CSF or M-CSF after 5 days and cells that received no further stimulus after 5 days (mock). In order to comprehensively analyze phenotypes of these MDM populations, we made use of an antibody panel previously used to characterize macrophage phenotypes in the context of renal cell carcinoma^16^ (Fig. S4A). The panel included 36 markers that clustered the differentially stimulated MDMs into M1-like and M2-like MDMs (Fig. 2A). CD38, CD274, CD197, CD54, CD82, CD86, and Slamf7 were expressed higher by M1-like MDMs (those stimulated with LPS, LPS + IFN-γ, and IFN-γ), whereas markers like CD163, CD206, and Neurophilin were expressed higher by M2-like MDMs (those stimulated with GC, GC + TGF-β, IL-4, and IL-10). Additionally CD87 was specifically upregulated by IC-treated MDMs, and CD64 was most highly expressed by IFN-γ-treated MDMs.

Next, we applied our previously described gating strategy (Fig. S1) to define differences in phagocytic affinity and capacity. We observed differences in phagocytic affinity and capacity that depended on stimulation conditions. The populations stimulated with IL-4 and IL-10 internalized up to 2-fold more *E. coli* cells than IC-, IFN-γ-, LPS-, LPS/INF-γ-, or GM-CSF-treated cells (Fig. 2B). GC− and GC + TGF-β-treated MDMs showed a phagocytic capacity intermediate between the prototypic M1 and M2 polarized conditions. A linear model to predict phagocytic capacity based on stimulation, replicate, and condition resulted in a good overall fit (R^2^ = 0.89) (Fig. S4B). However, we determined four outliers (Figs 2B and S4B). After outlier removal no significant replicate batch effect was detected anymore and the model fit increased to R^2^ = 0.96 (Fig. S4B). A pairwise comparison of all stimulations using a Tukey test revealed significant differences in osmium intensities between M1-like and M2-like polarized MDMs with the exception of the IC stimulation (Fig. S4C), which is considered an M2-like stimulus^33^. We also observed a significantly lower phagocytic capacity of GM-CSF-treated MDMs compared to M-CSF-treated MDMs, with the capacity of GM-CSF-treated cells comparable to that of M1-like MDMs (Figs 2 and S4C). Based on these results we conclude that M1-like stimuli (IFN-γ, LPS, LPS + IFN-γ) and GM-CSF reduce the capability of MDMs to efficiently phagocytosis *E. coli* cells relative to M-CSF-treated MDMs. On the other hand, M2-like stimuli (except IC) induce MDMs to be more efficient at scavenging and phagocytosing *E. coli* particles than M1-activated macrophages.

### Phagocytic affinity and capacity are correlated with particular marker expression patterns

**Figure 3:**
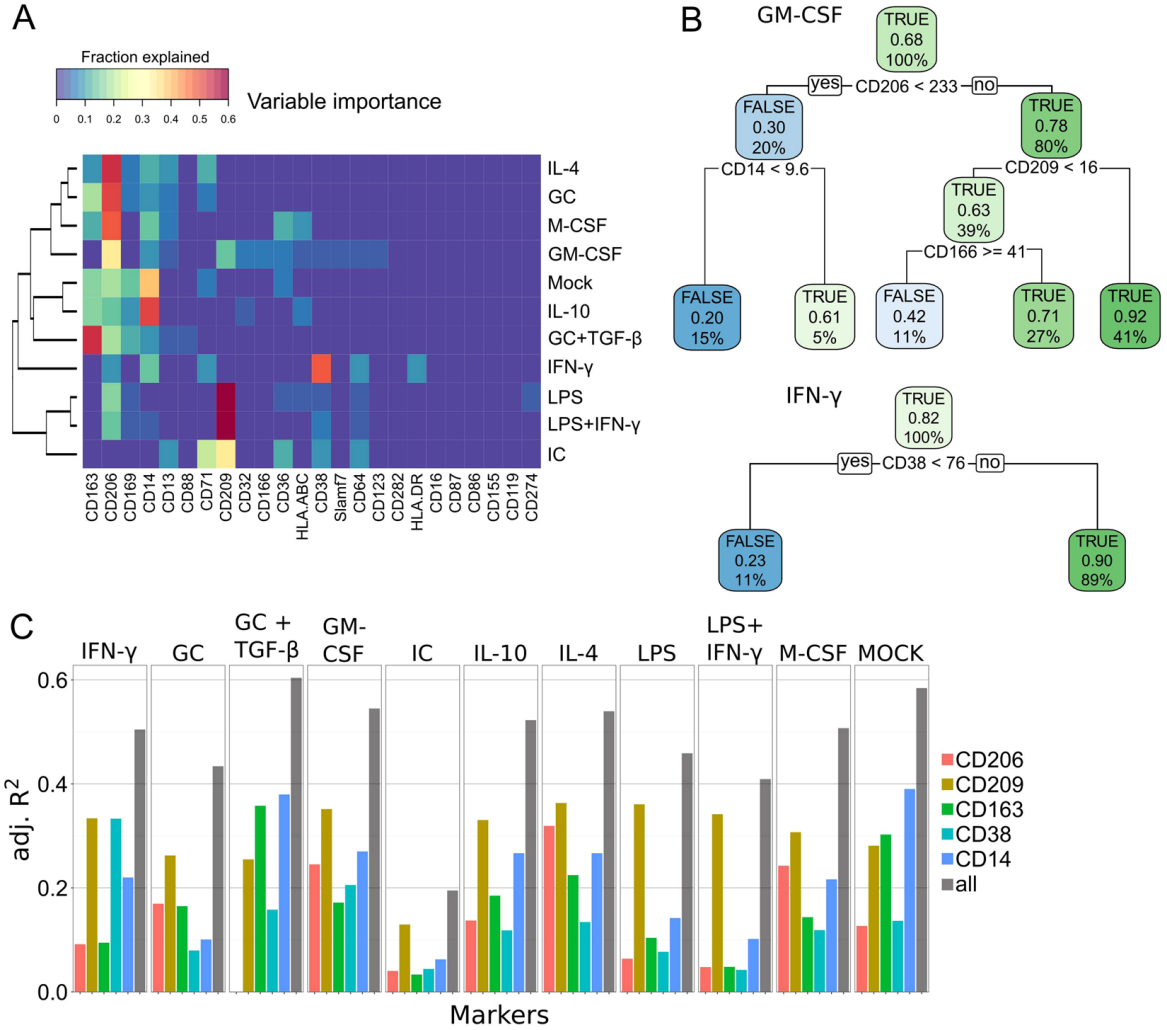
Markers associated with phagocytic affinity and capacity of MDM populations. (**A**) The importance of various markers across stimulations revealed by regression tree analyses are shown with the fractions of tree splits explained by each variable. The fractions in each row add up to 1. (**B**) Example regression trees for GM-CSF (top) and IFN-γ (bottom). Each node shows the predicted class (in phagocytosis gate TRUE/FALSE), the predicted probability of being in the phagocytosis-positive gate, and the percentage of events in the node. Each split is indicated by the marker and the value at which it was split. (**C**) Adjusted R^2^ values as a goodness of fit value for a linear regression model to predict the ^188^Os intensity as a function of CD206, CD209, CD163, CD38, or CD14 and as a function of all possible combinations of these markers. The conducted analysis contained cells of three biological and one technical replicate as in Fig. 2.

To identify makers correlated with phagocytosis of MDMs, we performed a regression tree-based analysis^34^ on our high-dimensional single-cell dataset. The regression tree was built by recursive partitioning during which markers were identified that best separated cells into phagocytic and non-phagocytic subgroups. For the MDM/T ratio 1:100, CD14, CD163, CD206, and CD209 were expressed on phagocytic MDMs with varying degrees of importance across the different MDM stimulations (Fig. 3A). Although single markers often explained most of the variance, combinatorial expression of multiple phagocytic markers identified the purest subgroups of phagocytic MDMs (Fig. 3B). For example, 78% of CD206^high^ MDMs had phagocytosed *E. coli* as compared to only 30% for CD206^low^ MDMs after GM-CSF stimulation. Using additional markers revealed that only 20% of CD206^low^ and CD14^low^ MDMs had phagocytosed *E*. *coli* as compared to 92% of CD206^high^ and CD209^high^ MDMs, indicating the importance of these proteins in phagocytosis. Additionally, only in GM-CSF-treated MDMs was CD166 negatively associated with phagocytosis and only in IFN-γ-treated MDMs was CD38 strongly associated with phagocytosis.

To systematically identify markers associated with phagocytic capacity, we first correlated the osmium intensity of MDMs with marker expression levels. This analysis revealed a fair correlation (R > 0.5) between the expression of CD209 and the osmium intensity in all stimulations except that with IC (Fig. S5), indicating that CD209 regulates the phagocytic capacity of MDMs. To our surprise none of the other markers in our panel were correlated with osmium intensity and thus phagocytic capacity. A linear regression model further highlighted that in most conditions CD209 was the strongest single determinant of phagocytic capacity (Fig. 3C); however, we also observed that the goodness of fit for the model increased when combinations of the previously identified five markers, CD14, CD38, CD163, CD206, and CD209, were considered (Fig. 3C). Our data suggest that only CD209 directly regulates the phagocytic capacity of MDMs while other markers more generally enable phagocytosis. Thus, the multiparametric approach revealed hierarchical dependencies of proteins associated with phagocytosis capability.

### Anti-CD47 antibody treatment increases phagocytosis of cancer cells by M1-like but not M2-like MDMs

We next investigated differences in phagocytosis of cancer cells by differently stimulated MDMs. To initialize phagocytosis, formaldehyde fixed and osmium-stained MDA-231 cells, which are a breast adenocarcinoma line, were added to MDMs polarized with M-CSF, LPS, IFN-γ, or IL4 for 2 hours, an incubation time that appeared reasonable given the high rate of phagocytosis observed for *E. coli*. Like other cancer cell lines, MDA-231 cells are known to express CD47^35^ and thus target cells were treated with an anti-CD47 antibody to assess antibody-dependent phagocytosis. Of note, the anti-CD47 antibody used in this study was previously shown to exert most of its positive effect on phagocytosis through blocking the SIRP-α “don’t eat me” signal on MDMs as opposed to stimulating Fc-receptor-dependent uptake of opsonized targets^36^. A representative gating strategy to identify phagocytic events is shown in Supplementary Fig. 6.

**Figure 4:**
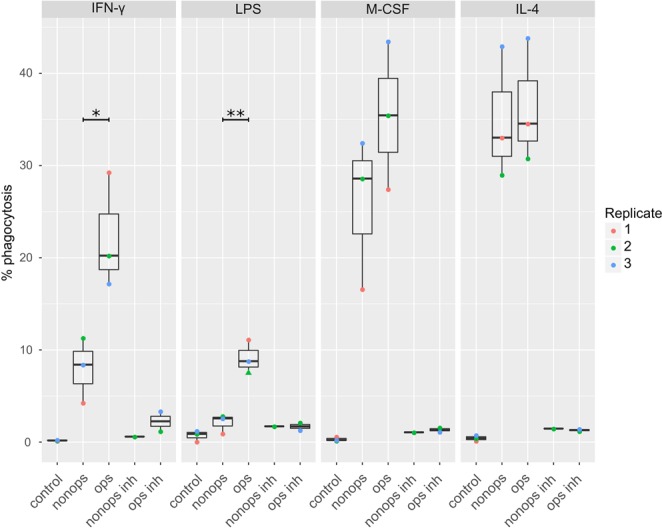
Phagocytosis of cancer cells by MDMs stimulated with IFN-γ, LPS, M-CSF, or IL-4. The percentages of MDMs in the phagocytosis gate are shown for up to three biological replicates (indicated by color). The controls were incubated without cancer cells. Abbreviations: nonops, addition of non-opsonized cancer cells; ops, addition of cancer cells that had been opsonized with anti-CD47 antibody; nonops inh, addition of non-opsonized cancer cells and cytochalasin D; and ops inh, addition of anti-CD47 opsonized cancer cells and cytochalasin D. In one replicate of LPS-stimulated MDMs incubated with opsonized cancer cells many MDMs were lost during the protocol, and the percentage shown here is only based on roughly 60 cells (indicated by triangle shape). This data point was excluded from the p-value calculations. Significances were calculated using the Student’s t-test (*p < 0.05; **p < 0.01).

As observed when *E. coli* cells were the targets, we observed different amounts of cancer cell phagocytosis by MDMs depending on the type of stimulation. When MDMs were not opsonized, those polarized with IL-4 and M-CSF more actively phagocytosed cancer cells than did LPS- and IFN-γ-stimulated MDMs (Fig. 4, compare “non-ops” lanes). We next compared phagocytosis of target cells that had been opsonized with an anti-CD47 antibody. Although we observed similar levels of phagocytosis-positive MDMs stimulated with IL-4 or M-CSF with or without opsonization, a significant increase in phagocytosis of opsonized compared to non-opsonized targets was observed for MDMs stimulated with LPS (p-value 0.007) and with IFN-γ (p-value 0.027; Fig. 4, compare “ops” lanes). No markers in our antibody panel correlated with efficiency of cancer cell phagocytosis. We concluded that our metal-based phagocytosis assay can be used to detect differential phagocytosis of cancer cells by macrophages and found that anti-CD47 opsonization increased phagocytosis by M1-like MDMs.

## Discussion

Here were demonstrated that our novel mass cytometry-based protocol enables studies of phagocytosis in vitro. Osmium tetroxide and ruthenium tetroxide were used to fix and stain E. coli or cancer cells, and these cells inside or bound to MDMs were detected simultaneously with markers known to reveal the phenotypic diversity of macrophages by mass cytometry. This novel framework allowed in-depth characterization of phagocytes and their phagocytic activity and revealed receptor expression patterns linked to phagocytic affinity and capacity.

Phagocytosis of *E. coli* cells was rapid. After 30 minutes 60–90% of MDMs, depending on stimulus, had phagocytosed osmium-labeled target cells. Treatment with cytochalasin D, a potent phagocytosis inhibitor that interferes with actin polymerization^31^, almost completely blocked phagocytosis, validating our assay. Interestingly, at an MDM to target cell ratio of 1 to 50, differentially stimulated MDMs had similar phagocytic affinities: that is the percentage of MDMs in a population that phagocytosed a target cell was similar. In contrast, the phagocytic capacities, reflecting the number of target cells engulfed, differed. Only under limiting target cell ratios (1/10) the differences in phagocytic affinities of MDMs treated with certain stimuli became apparent.

Most stimuli induced the emergence of phenotypically distinct macrophage populations. While we found some overlap between marker expressions that characterize certain populations with a study from Roussel *et al*.^23^, we also observed differences. The most prominent was in expression of PD-L1, which was most strongly expressed in M1-like conditions in our hands but in M2-like conditions by Roussel *et al*. This was likely due to differences in stimulation protocols. Taking phagocytosis as a measure of function, these diverse stimulations were reflective of the classical M1/M2 model with M2-like MDMs showing higher phagocytic activity. These findings are also in agreement with previous studies that showed that IL-10-stimulated monocytes have enhanced phagocytosis capability compared to unstimulated cells^26^, that IL-4- and GC-stimulated MDMs are more active than IFN-γ-stimulated MDMs^27^, and that human macrophages educated by IL-10 and IL-4 more rapidly take up mycobacteria than M1-like cells^37^.

Using a recursive partitioning algorithm and a linear modeling approach we further disentangled the importance of single markers for phagocytic affinity and capacity across all stimulations. Our antibody panel contained markers belonging to the scavenger receptor class (CD36, CD68, CD163, and CD204), pattern recognition receptors (CD14, CD206, CD209, and CD282), and receptors of the complement system (CD11b, CD16, CD32, CD64, and CD88). Our correlation analysis indicated that for the ingestion of non-pathogenic *E. coli* DH5-*α* cells, pattern recognition receptors and the scavenger receptor CD163 were important. CD14 is known to be involved in the binding of LPS and phagocytosis of *E. coli* by human monocytes and macrophages^26,38^, and the importance of CD209 in LPS binding and phagocytosis of *E. coli* was described for human dendritic cells^39,40^. Additionally, we showed that CD206 expression and CD163 expression are associated with phagocytosis. CD206^41^ and CD163^42^ are known to be involved in phagocytosis of numerous strains of bacteria, which further strengthens the confidence in our analysis approach. Based on our analysis, CD209 seems most strongly associated with phagocytic capacity, whereas other markers were associated with phagocytosis competence. As the importance of particular markers varied with the polarization, only a multiplexed approach, as presented here, is capable to evaluate the importance of individual markers.

We observed that IFN-γ stimulation strongly upregulated CD38, which in turn was associated with phagocytosis capacity. The association of CD38 with phagocytosis may result from a function of CD38 in Ca2+ release, which may facilitate efficient particle ingestion through a mechanism similar to that described for Fcγ receptor-dependent phagocytosis^43^. Our analysis also revealed that CD166 expression is negatively associated with phagocytic activity. CD166 is involved in cell-to-cell adhesion and is localized at endothelial cell junctions, where it may act as a cell density sensor and has been shown to control tissue invasion in melanoma progression^44^. It has been reported that MDMs differentiated in higher cell densities show less phagocytic activity^45^. We therefore suspect that CD166 and phagocytic activity are linked via cell density effects.

To our surprise the expression of CD204, a macrophage scavenger receptor, was not associated with phagocytosis capacity. By analysis of the effects of deletion of *CD204* in mouse cells, CD204 was shown to be important for the uptake of certain *E. coli* strains, including DH5-α^46^. We speculate that we did not observe a correlation with CD204 here due to the greater importance of other receptors in wild-type macrophages and potential differences between mouse and human.

Our multiparametric approach distinguishes itself for its ability for fast screening of molecular components related to phagocytosis while revealing inherent hierarchical dependencies. Going forward, further targeted validation experiments need to be conducted to confirm the causative relationship of the identified proteins with phagocytosis beyond the correlative nature described here.

Finally, we showed that our mass cytometry method can also be used to quantify Fc-receptor-mediated phagocytosis of cancer cells. Monoclonal antibody treatment is a common strategy used in cancer therapy^47^, and macrophages are known to be involved in the mechanism of antibody-dependent phagocytosis that underlies efficacy of this treatment^25,29,48,49^. Studying Fc receptor-mediated phagocytosis is therefore of high clinical relevance and enhancing tumoricidal activity of macrophages by drug treatment represents an attractive therapeutic strategy. Similar to the results obtained in our analysis of *E. coli* phagocytosis, we observed more phagocytosis by IL-4- and M-CSF-stimulated MDMs with and without opsonization than by IFN-γ- and LPS-stimulated MDMs. Strikingly, phagocytosis capacity of IL-4- and M-CSF-stimulated MDMs was not increased upon opsonization with anti-CD47 antibody treatment, whereas IFN-γ- and LPS-stimulated MDMs phagocytosis capacity was significantly increased by opsonization. At least for IFN-γ, this could be explained by high expression levels of the FcγRI receptor CD64.

Contradictory to our findings, a previous study observed higher amounts of phagocytosis by M1-like MDMs (IFN-γ or LPS stimulated) than by M2-like MDMs^50^. However, Zhang *et al*. also observed that M1-like macrophages were more responsive to anti-CD47 antibody treatment than were M2-like MDMs and that the phagocytic activity varied strongly depending on target cell line. Additionally, it should be noted that there were differences in the stimulation protocols used by Zhang *et al*. and by us. In a mouse study using yet another stimulation protocol, opsonization led to a significant increase in phagocytosis by M-CSF-treated MDMs^25^. We conclude that, at least *in vitro*, differentially stimulated MDM populations respond differently to anti-CD47 antibody treatment and further studies are needed to elucidate the mechanisms that render some macrophages susceptible to “don’t eat me” signals and to opsonization. We would like to note that the protocol presented here simultaneously stained the phagocytosed cancer cells and the MDMs. This hindered the discovery of markers associated with phagocytosis but could potentially allow for the definition of preferred targets of phagocytosis out of a complex mix of targets through target cell barcoding and detection of the ingested targets in future experiments.

In conclusion, using this novel assay we showed strong evidence for a differential phagocytic activity of M1-like and M2-like MDMs toward *E. coli* and cancer cell targets. We found that M2-like MDMs had higher phagocytic affinity under low target cell densities and higher phagocytic capacity under high target cell densities than did M1-like MDMs. The assay presented here could be easily adapted to detect other, non-toxic agents that bind to target cells such as iodouridine or cisplatin to circumvent target cell fixation and osmium tetroxide staining. Furthermore this high-dimensional approach could be used to study signaling responses in single cells after phagocytosis of different targets. It would also be interesting to investigate how the phenotypes of phagocytes change upon prolonged phagocytosis.

## Material and Methods

### Monocyte isolation from buffy coat

Human buffy coat was obtained from healthy anonymized donors at the Bloodbank (Blutspendezentrum) Zurich. Human peripheral blood mononuclear cells (PBMCs) were isolated from buffy coat based on density Ficoll gradient centrifugation. The buffy coat was diluted in PBS and underlaid with histopaque-1077. For separation of the various cell fractions the buffy coat-histopaque emulsion was centrifuged for 30 min at room temperature at 450 g. PBMCs at the interface were collected, washed once in PBS at 4 °C and resuspended in PBS and counted using a Neubauer cell counting chamber. Monocytes from human PBMCs were isolated using the Pan Monocyte Isolation Kit (Miltenyi Biotec GmbH) according to the manufacturer’s instructions. Viable monocytes were counted using a Neubauer chamber, and resuspended in RPMI-1640 supplemented with 10% DMSO for cryo-preservation in liquid nitrogen.

### Differentiation and stimulation

To differentiate peripheral blood monocytes *in vitro* into macrophages we followed the guidelines proposed by Murray *et al*. Monocytes were stimulated for 5 days with 30 ng/ml macrophage-colony-stimulating factor (M-CSF) in RPMI 1640 media. Cells were seeded in 6-well tissue culture plates (Thermo Fisher Scientific) at a density of 0.5 × 10^6^ cells per ml in 2.5 ml per well. The monocytes were allowed to adhere and differentiated into MDMs for 5 days under a 5.0% CO_2_ atmosphere at 37 °C. On the fifth day the original media was removed and replaced with 2.5 ml/well RPMI 1640 supplemented with different stimulatory agents (Table 1). No media change after 5 days was performed on mock samples. To create an antigen-antibody complex for macrophage stimulation, 25 µl anti-chicken egg albumin antibody (whole antiserum) was mixed with 1.66 µl ovalbumin (10 mg/ml) and incubated at 37 °C for 1 h. MDMs were polarized for 24 h prior to phagocytosis assays.

**Table 1:** MDM stimulation agents.

Stimulus	Final concentration	Manufacturer
M-CSF	30 ng/ml	PeproTech
GM-CSF	30 ng/ml	PeproTech
IFN-ɣ	0, 1 µg/ml	PeproTech
LPS	0, 1 µg/ml	Sigma-Aldrich
IFN-ɣ + LPS	0, 1 µg/ml + 0,1 µg/ml	PeproTech and Sigma-Aldrich, respectively
IL-4	20 ng/ml	PeproTech
IL-10	10 ng/ml	PeproTech
GC (dexamethasone)	40 ng/ml	Sigma-Aldrich
GC + human recombinant TGF-β	10 ng/ml	Sigma-Aldrich and Cell Signaling Technology, respectively
antigen-antibody complex (anti-chicken egg albumin antibody and ovalbumin)	10 µg/ml	Sigma-Aldrich

### Target cell preparation and metal-based staining

For *E. coli* staining, 5 ml of Ty-Medium were inoculated with *E. coli* strain DH5-α and incubated overnight at 37 °C under constant shaking (250 rpm). Several *E. coli* overnight cultures were pooled, washed once with PBS, and the cell number was determined by optical density (BioPhotometer Plus, Eppendorf). It was assumed that an OD_600_ of 1 equals 8 × 10^8^ *E. coli* cells per ml solution, and 1 × 10^9^ cells were subsequently fixed in 1 ml of a 1.6% paraformaldehyde in PBS for 10 min at room temperature. After fixation *E. coli* cells were washed twice with PBS and resuspended in 1 ml PBS per 2 × 10^9^ cells. A 2x stock of the PBS/metal-staining solution was quickly mixed and vigorously vortexed with the prepared cell suspension (1:1). The final staining concentrations in 1 ml PBS for OsO_4_ and RuO_4_ were 0.00008% (w/v) and 0.00015% (w/v), respectively, per 1 × 10^9^ *E. coli* cells. The mixture was allowed to react for 7 min at room temperature and centrifuged for 3 min at 3200 g. The metal-labeled *E. coli* cells were washed twice with PBS, resuspended in PBS and filtered through a 40-µm cell filter for the removal of larger particles. Cell numbers were determined based on optical density and labeled *E. coli* cells were aliquoted, pelleted, and stored at −20 °C.

Human MDA-MB-231 cells were obtained from ATCC and cultured in Leibovitz L15 medium supplemented with 10% FBS at 37 °C. Cells were washed once using PBS, detached using 0.25% trypsin/EDTA, and fixed using 1.6% PFA. Fixed cell pellets were stored at −80 °C. For osmium staining, cells were resuspended in PBS, and cell numbers were determined using Countess (ThermoFisher). For staining, 1 × 10^6^ cell were incubated in 1 ml of a 1:500’000 dilution of OsO_4_ in PBS for 7 min. Cells were centrifuged at 1000 g for 3 min, the supernatant was discarded, and the pellet was washed twice with PBS. Pellets were resuspended in PBS, and samples were filtered through a 40-µm cell filter. Stained cells numbers were counted, and cells were resuspended at desired concentrations for subsequent use.

### Antibody conjugation

Metal-labeled antibodies used in this work, including manufacturer and staining-concentrations, are listed in Table 2. Unconjugated antibodies were metal tagged using the MaxPar Antibody Labeling kit (Fluidigm) according to the manufacturer’s protocol. Prior to use, the final concentration of each metal-labeled antibody was individually determined by titration and subsequent CyToF measurements. For long-term storage conjugated antibodies were diluted in antibody stabilization solution (Candor Bioscience GmbH) and stored at 4 °C.

**Table 2:** Antibodies used for mass cytometry staining.

Number	Antibody	Clone	Metal	conc [µg/ml]
1	CD81	5A6	Yb173	0.12
2	CD169	7-239	Er170	0.25
3	CD32	FUN-2	Er166	0.25
4	HLA-ABC	W6/32	Yb172	0.25
5	PD-L1	29E.2A3	Gd160	0.25
6	CD304	12C2	Gd156	0.5
7	CD38	HIT2	Nd142	0.5
8	CD54	HA58	Yb176	0.5
9	CD82	ASL-24	Tb159	0.5
10	CD86	2331 (FUN-1)	Yb171	0.5
11	CD88	S5/1	Yb174	0.5
12	CD14	RMO52	Er168	1
13	CD163	GHI/61	Tm169	1
14	CD204	351615	Sm149	1
15	CD206	15-2	Sm147	1
16	CD64	10.1	Pr141	1
17	CD68	Y1/82 A	Nd143	1
18	CD71	CY1G4	Nd145	1
19	CD87	VIM5	Dy163	1
20	HLA-DR	L243	Lu175	1
21	CD119	GIR-208	Dy161	2
22	CD11b	M1/70	Eu151	2
23	CD120b	3G7A02	Dy164	2
24	CD123	6H6	Nd150	2
25	CD13	WM15	Nd148	2
26	CD155	SKII.4	Nd146	2
27	CD16	3G8	Er167	2
28	CD166	3A6	Gd158	2
29	CD197	GO43H7	Sm154	2
30	CD209	DCS-8C1	La139	2
31	CD282	TL 2.1	Ho165	2
32	CD36	5-271	Nd144	2
33	CD40	5c3	Sm152	2
34	CXCR4	12G5	Dy162	2
35	PD-L2	MIH18	Gd155	2
36	SLAMF7	162.1	Eu153	2

### Mass cytometry-based phagocytosis assay

The CyToF-based phagocytosis assay was conducted with 24-h pre-stimulated MDMs and metal-labeled target cells. For experiments with *E. coli*, frozen aliquots of metal-labeled cells were thawed and 1 × 10^6^ cells/µl were resuspended in PBS, vortexed, and stored on ice until use. Labeled MDA-MB-231 cells were resuspended in 50 µl RPMI-1640 (room temperature, containing 10% FBS) per 1.25 × 10^9^ cells. If used, cytochalasin D (5 µM) was applied to the selected wells 10 min prior to target cell addition (of note, addition of Cytochalasin D 60 min prior to target cell addition, as done for the RuO_4_ experiments, led to lower levels of *E. coli* uptake inhibition potentially due declining inhibitor effects). For cancer cell opsonization, fixed and OsO_4_ stained cancer cells were diluted in RPMI media at 10 × 10^6^ cells per ml and then incubated with 0.05 µg of anti-CD47 antibody (B6H12, eBioscience) per 1 mio cells for 30 min at 37 °C. Cells were then pelleted, supernatant discarded and resuspended in RPMI media at a concentration of 2.5 mio/ml. If not stated otherwise, labeled *E. coli* were added in a 1:10 or 1:100 MDM/T ratio per well and cancer cells at a 1:2 ratio. After target cells were added to the MDMs, plates were moderately shaken to ensure that target cells were spread throughout the entire well. Macrophages were then allowed to phagocytose *E. coli* for 30 min or MDA-MB-231 cells for 2 h. After incubation the supernatant was removed, and dead cells were stained with cisplatin. Thereafter, 2 ml of a 1:1000 cisplatin/PBS solution were added per well with a multi-dispense pipette. Cells were stained for 60 s at room temperature, and then the supernatant was removed. Each well was then washed with 2 ml PBS at room temperature to ensure that all loosely attached target cells were removed. Cells were then fixed with 2 ml 1.6% PFA/PBS per well for 10 min. After incubation the fixation solution was replaced with 2.5 ml PBS per well and macrophages were gently scraped from the plastic surface with small cell scrapers. Macrophage suspensions were transferred into 15-ml centrifugation tubes on ice. Samples were centrifuge at 1000 g for 2 min at 4 °C, and then the supernatant was removed and cells were resuspended in 250 µl chilled cell staining media (CSM; PBS, 0.5% BSA, 2 mM EDTA). Samples were transferred into wells of a 96-well plate and centrifuged (1000 g, 2 min, 4 °C), and the supernatant was removed. Cell pellets were disrupted and the sample plate was (optionally) stored at −80 °C prior to barcoding.

### Barcoding and acquisition

Palladium- and lanthanide-based mass tag cell barcoding was performed prior to metal-labeled antibody staining to allow comparable antibody staining^51^. Six palladium isotopes (^102^Pd, ^104^Pd, ^105^Pd, ^106^Pd, ^108^Pd, ^110^Pd) were conjugated with bromoacetamidobenzyl-EDTA (BABE) and two indium isotopes (^113^In and ^115^In) were conjugated to 1,4,7,10-tetraazacy-clododecane-1,4,7-tris-acetic acid 10-maleimide ethylacetamide (mDOTA) following standard procedures^52^. Samples were temporarily permeabilized with a 0.03% saponin-PBS solution containing four unique combinations of 100 nM of the barcoding reagents and incubated 1 h at room temperature^53^. The barcoded samples were washed twice with 3 mg/ml PBS-saponin and twice with CSM and then pooled for subsequent antibody staining. Approximately 12 × 10^6^ cells were stained in 100 µl of CSM antibody solution (see Table 2 for antibody concentrations) for 30 min at 4 °C. Following the incubation, stained samples were washed twice with CSM and then once with PBS. To stain cells for DNA, cells were resuspended and incubated overnight in 1.6% PBS/paraformaldehyde containing iridium DNA intercalator (Fluidigm, final concentration 5 pM). On the next day samples were washed twice with PBS and twice with ddH_2_O, resuspended in ddH_2_O, and filtered through a 70-µm cell strainer. Samples were mixed with Four Element Calibration Beads (Fluidigm) in a 1:10 (v/v) ratio. Samples were analyzed on a CyTOF 1 mass cytometer (*E. coli* assays, 1 replicate of cancer cell assay) and a Helios (2 replicates of cancer cell assay) (Fluidigm).

### Data processing and visualization

Acquired .fcs files were concatenated using the Cytobank concatenation tool, normalized based on the calibration beads (Fluidigm) with an executable Matlab-based tool of the Normalizer tool^54^, and then de-barcoded using a Matlab-executable single-cell debarcoder tool^55^. The fcs files were uploaded into Cytobank for manual gating, scatter plot display, and export of gated populations for further analysis. For *E. coli* experiments, gates to identify MDMs were set to exclude cell debris (^191^Ir and ^193^Ir gate) followed by CD68^+^ cell selection. For the cancer cell assay MDMs were detected either as CD68 and CD206 double-positive population or as the CD68 and HLA-DR double-positive population. Phagocytic events were detected within the MDM gate as positive for ^188^Os.

### Statistical analysis and modeling

Statistical data analysis and modelling was conducted in R (R Development Core Team)^56^ and plotted using ggplot2 package^57^. A linear regression model was used to predict the ^188^Os median intensity of each MDM/T 1:100 sample based on stimulation and replicate (Fig. S4). From the regression model 4 of 49 samples were defined as outliers as judged from QQ plots and were excluded from calculations of statistical significances as well as multivariate marker analysis (Fig. S4). Statistically significant differences between ^188^Os intensities of stimulations were calculated in a Tuckey post hoc test after removal of four outliers as previously identified in the regression model (Fig. S4). Multivariate analysis of phagocytotic marker patterns was performed using the Recursive PARTitioning algorithm (rpart) implementation for R on arcsinh transformed data^58^. Classification trees were trained on two replicates (minimal terminal node size = 5% of sample size; cp = 0.01; crossvalidation = 10) and variable importances and classification trees were retrieved from these two replicates. The prediction accuracy was estimated by predicting phagocytic cells in the remaining two replicates. Depending on the stimulation this resulted in prediction accuracy between 76% and 99%.

Linear regression to predict ^188^Os levels from marker expression was performed on the five most predictive markers for phagocytosis (CD14, CD38, CD163, CD206, CD209) as defined by the classification tree analysis. Adjusted R^2^ to predict ^188^Os intensities from marker expression were calculated for each marker alone and a combination of all five of the above markers.

## Supplementary information

Supplementary Dataset 1

## Data Availability

The datasets generated during the current study are available in Cytobank (https://community.cytobank.org/cytobank/projects/1208). R scripts for data analysis are available upon request from the corresponding author.
