# Supplementary material for: In-Depth Characterization of Monocyte-Derived Macrophages using a Mass Cytometry-Based Phagocytosis Assay
Source: Sci Rep. 2019 Feb 13;9:1925. doi: 10.1038/s41598-018-38127-9 (PMC6374473; doi:10.1038/s41598-018-38127-9)
Supplement: Supplementary file 1 — Supplementary Dataset 1 [file 41598_2018_38127_MOESM1_ESM.pdf]

Supplementary Information

In-Depth Characterization of Monocyte-Derived Macrophages using a Mass Cytometry-Based Phagocytosis Assay

Daniel Schulz[1,4], Yannik Severin[2,4], Vito Riccardo Tomaso Zanotelli[1,3] and Bernd Bodenmiller[1,5,*]

[1]Institute of Molecular Life Sciences, University of Zürich, Winterthurerstrasse 190, 8057 Zürich, Switzerland

[2]Department of Biology, Institute of Molecular System Biology, Swiss Federal Institute of Technology (ETH Zürich), Otto-Stern-Weg 3, Zürich, Switzerland.

[3]Systems Biology PhD Program, Life Science Zürich Graduate School, ETH Zürich and University of Zürich, 8057 Zürich, Switzerland

[4]These authors contributed equally

[5]Lead contact

*Correspondence: bernd.bodenmiller@imls.uzh.ch

Figure S1

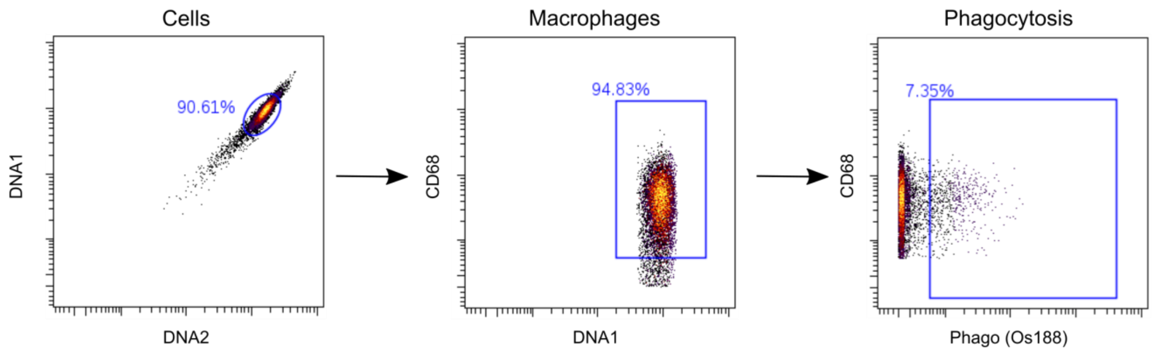

Figure S1: Gating scheme to identify cells that have phagocytosed OsO_4-labeled *E. coli*. A DNA gate was set to select cells. The MDMs were then selected by CD68 expression, and phagocytosis was determined based on a global, manually defined gate for ^{188}Os intensity.

Figure S2

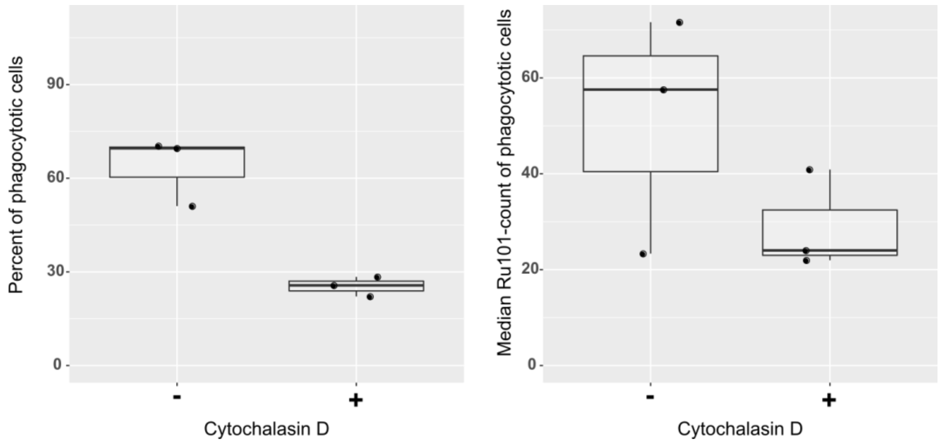

Figure S2: Proof of concept for RuO_4-based phagocytosis detection. M-CSF-treated MDMs were treated or not with cytochalasin D for 60 min prior to target cell addition. *E. coli* target cells were added, and samples were incubated for 30 min. Data are from three replicates.

Figure S3

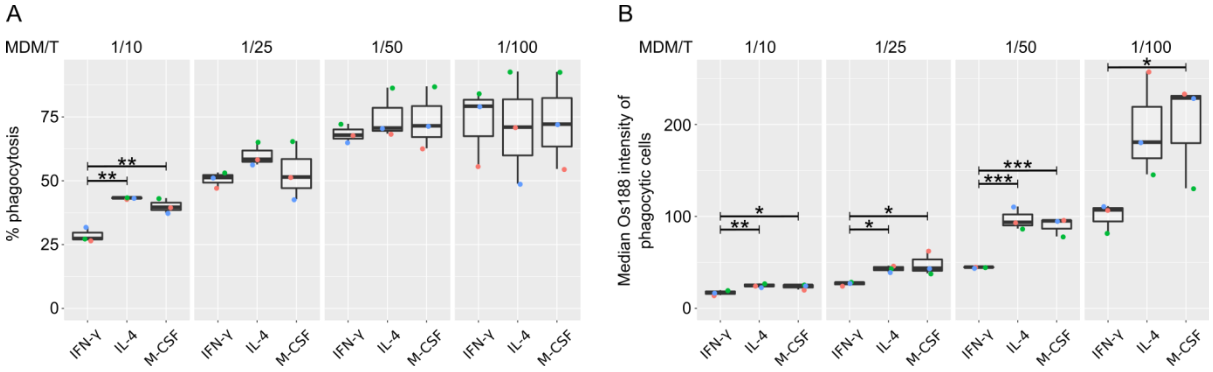

Figure S3: Phagocytic capacity and affinity of MDMs at indicated MDM to target cell ratios. A) The percentages of phagocytosis-positive cells are shown for three different MDM stimulations and four different ratios of MDMs to *E. coli* cells. Data are from three replicates. B) The median intensities of ^{188}Os in the phagocytosis gate for indicated stimulations and different MDM to target ratios. Significances were calculated using the Student's t-tests (*, $p < 0.05$;**, $p < 0.01$; ***, $p < 0.001$).

Figure S4

A

Scavenger receptors	Costimulatory molecules	Opsonic and complement receptors
CD163	CD155	CD32
CD68	CD54	CD64
CD36	PD-L2	CD11b
CD204	PD-L1	CD88
Adhesion molecules	CD40	CD16
CD169	SLAMF7	Cytokine receptors
CD166	CD86	CD119
Cd82	HLA-ABC	CD123
CD81	HLA-DR	CD120b
Metalloprotease	Patter recognition receptors	CD71
CD13	CD206	CD304
ecto-enzyme	CD14	Chemokine receptors
CD38	CD282	CD197
CD87	CD209	CXCR4

B

Model: $\log(\text{Intensity}_i) = \beta_1 \times \text{Stimulation} + \beta_2 \times \text{Replicate} + \beta_3 \times \text{Condition} + \varepsilon_i$

Model summary

Multiple R-squared: 0.89

F-statistic: 39.77 on 13 and 66 DF, p-value < 2.2e-16

	Df	Sum Sq	Mean Sq	F value	Pr (>F)	
Stimulation	9	3.348	0.372	3.6493	0.0009525	***
Replicate	3	1.984	0.661	6.4885	0.0006478	***
Condition	1	47.362	47.362	464.6779	< 2.2e-16	***
Residuals	66	6.727	0.102			

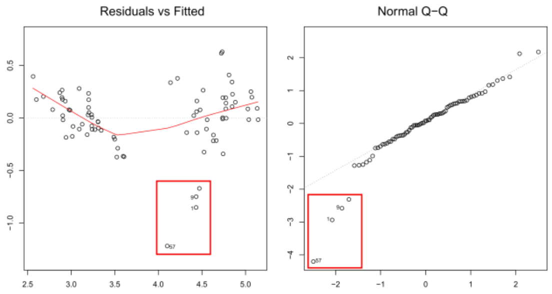

Model summary after removing four outliers

Multiple R-squared: 0.96

F-statistics: 130.4 on 13 and 62 DF, p-value < 2.2e-16

	Df	Sum Sq	Mean Sq	F value	Pr (>F)	
Stimulation	9	3.128	0.348	10.4924	1.16e-09	***
Replicate	3	0.231	0.077	2.3237	0.08361	.
Condition	1	52.804	52.804	1593.8991	< 2.2e-16	***
Residuals	62	2.054	0.033			

C

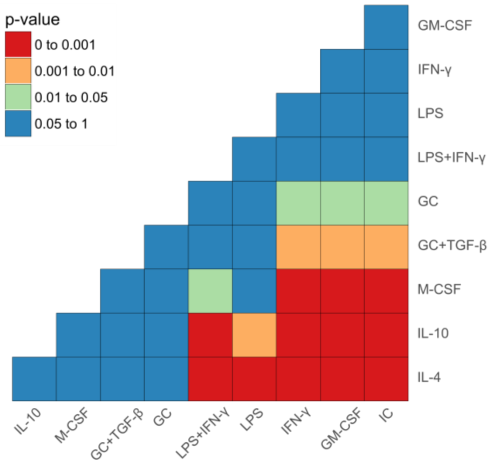

Figure S4: A) Antibody panel used in this study. B) Linear regression model used to predict the ^{188}Os intensity based on stimulation, replicate and condition (1to10 and 1to100). From the diagnostic plots four samples were defined as outliers indicated in the red boxes. The model summary is shown for the model before and after removing the outliers. C) Heatmap of Tukey HSD post hoc analysis of significant differences in phagocytosis for the different stimulations.

Figure S5

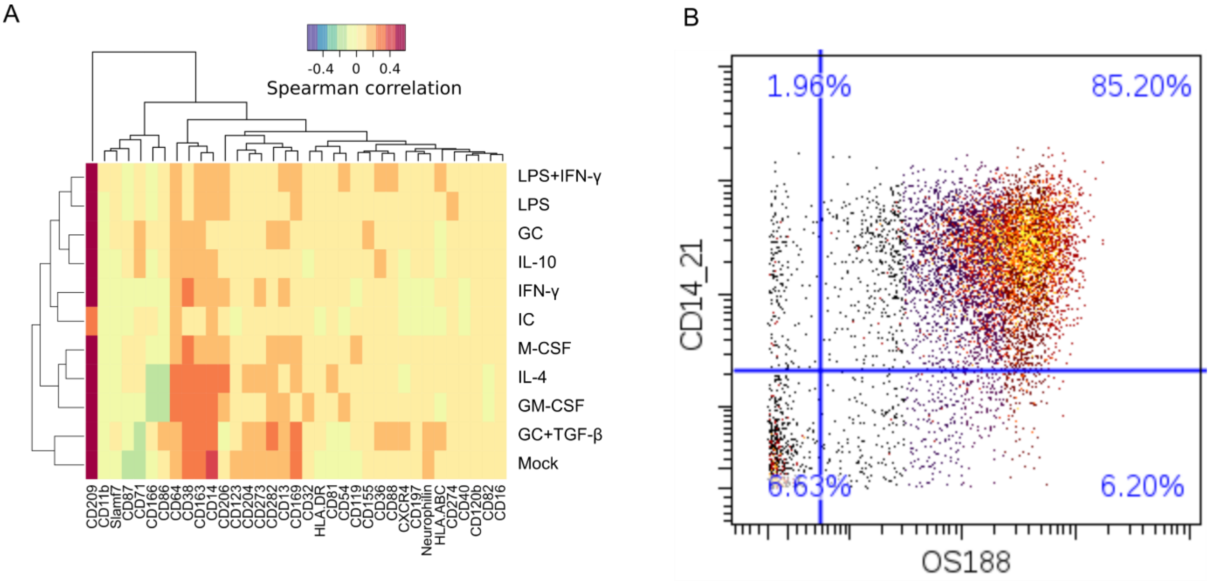

Figure S5: Markers associated with phagocytosis. A) Heatmap showing the Spearman correlation of each marker with ^{188}Os intensity across all cells from the phagocytosis-positive gate. B) Scatterplot showing how CD14^{low} cells are unlikely to phagocytose *E. coli* cells compared to CD14^{high} cells.

Figure S6

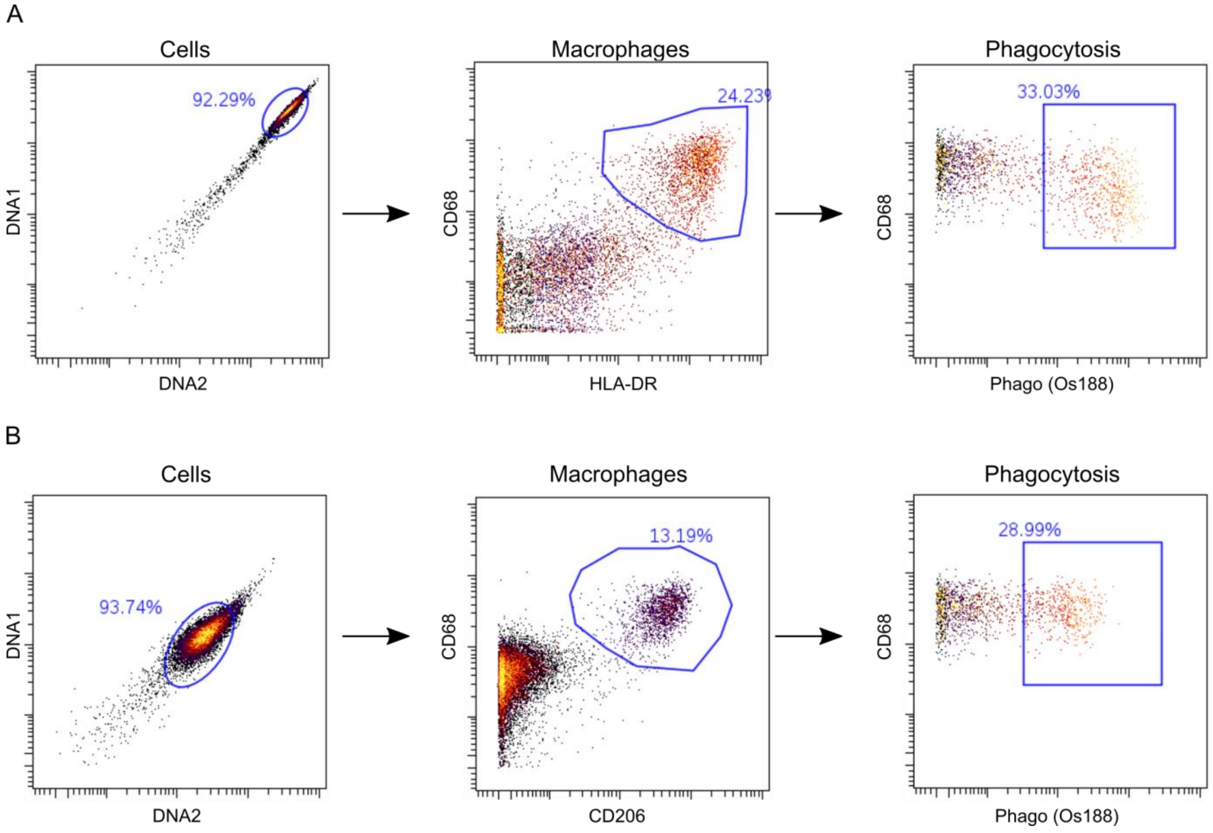

Figure S6: Gating strategy to identify MDMs that phagocytose cancer cells. A) In replicate 1 a DNA gate was used to select cells. Macrophages were thereafter defined as cells that were $CD68^+$ and HLA-DR^+. Phagocytosis was quantified based on a global, manually defined gate for ^{188}Os intensity. B) In replicates two and three the HLA-DR stain did not work. Therefore after defining cells, macrophages were defined as cells that were $CD68^+$ and $CD206^+$. Phagocytosis was quantified based on a global, manually defined gate for ^{188}Os intensity.
